# Comparison of methods for identifying causative bacterial microorganisms in presumed acute endophthalmitis: conventional culture, blood culture, and PCR

**DOI:** 10.1186/s12879-017-2264-5

**Published:** 2017-02-21

**Authors:** Pear Pongsachareonnont, Worawalun Honglertnapakul, Tanittha Chatsuwan

**Affiliations:** 1 Department of Ophthalmology, Faculty of Medicine, Chulalongkorn University; and King Chulalongkorn Memorial Hospital, Thai Red Cross Society, Bangkok, Thailand; 2 Department of Microbiology, Faculty of Medicine, Chulalongkorn University, Bangkok, Thailand

**Keywords:** Bacterial detection, Presumed acute endophthalmitis, Conventional culture, Blood culture, Polymerase chain reaction

## Abstract

**Background:**

Identification of bacterial pathogens in endophthalmitis is important to inform antibiotic selection and treatment decisions. Hemoculture bottles and polymerase chain reaction (PCR) analysis have been proposed to offer good detection sensitivity. This study compared the sensitivity and accuracy of a blood culture system, a PCR approach, and conventional culture methods for identification of causative bacteria in cases of acute endophthalmitis.

**Methods:**

Twenty-nine patients with a diagnosis of presumed acute bacterial endophthalmitis who underwent vitreous specimen collection at King Chulalongkorn Memorial Hospital were enrolled in this study. Forty-one specimens were collected. Each specimen was divided into three parts, and each part was analyzed using one of three microbial identification techniques: conventional plate culture, blood culture, and polymerase chain reaction and sequencing. The results of the three methods were then compared.

**Results:**

Bacteria were identified in 15 of the 41 specimens (36.5%). Five (12.2%) specimens were positive by conventional culture methods, 11 (26.8%) were positive by hemoculture, and 11 (26.8%) were positive by PCR. Cohen’s kappa analysis revealed *p*-values for conventional methods vs. hemoculture, conventional methods vs. PCR, and hemoculture vs. PCR of 0.057, 0.33, and 0.009, respectively. Higher detection rates of *Enterococcus faecalis* were observed for hemoculture and PCR than for conventional methods.

**Conclusions:**

Blood culture bottles and PCR detection may facilitate bacterial identification in cases of presumed acute endophthalmitis. These techniques should be used in addition to conventional plate culture methods because they provide a greater degree of sensitivity than conventional plate culture alone for the detection of specific microorganisms such as *E. faecalis.*

**Trial registration:**

Thai Clinical Trial Register No. TCTR20110000024.

## Background

Infectious endophthalmitis is a rare but serious disease with sight-threatening complications. The prognosis of patients with endophthalmitis depends on various factors, including the patient’s baseline condition, the source of infection, the severity of clinical symptoms, and the causative bacterial pathogen [1–3]. The common causative pathogens of acute bacterial endophthalmitis vary depending on geographic location and on the specific infection source. In cases associated with post-cataract surgery, coagulase-negative staphylococci are commonly isolated [4], while *Bacillus* species are predominantly found in post-traumatic endophthalmitis cases [1]. Several other conditions mimic the clinical presentation of endophthalmitis, including ocular inflammation from non-infectious uveitis, fungal endophthalmitis, and toxic anterior segment syndrome; however, bacterial cultures are negative in these cases. Identification of the causative bacterial pathogens in cases of acute bacterial endophthalmitis increases the likelihood of successful treatment because appropriate antibiotics can be selected.

The rate of positive bacterial identification in cases of endophthalmitis is 44.4–46% using conventional culture methods, in which the specimen is directly applied onto nutrient agar and incubated to facilitate the growth of bacteria [5, 6]. Rates of identification increase to approximately 50–70% when hemoculture bottles are used [7–11]. Hemoculture has the additional advantages of standardized preparation, relatively low specimen volume requirement, convenient transportation to the laboratory, and increased availability in rural areas. However, this method has some limitations, including the need for at least 0.1 ml of specimen, the requirement for specific equipment, and an inability to detect microorganisms other than bacteria. PCR followed by gene sequencing has the highest rate of detection, with positive identification in approximately 63–95% of bacterial endophthalmitis cases [12–19]. The PCR and sequencing approach requires only a small amount of specimen and usually results in rapid identification, but the equipment required can be costly. Additionally, this method produces a high rate of false-positive results. Currently, there is no consensus as to which of these bacterial detection techniques should be included in routine clinical practice, and no previous studies have compared the results of hemoculture with those of PCR-based identification.

The purpose of this study was to compare the efficacy of bacterial identification techniques (conventional plate culture, the VersaTrex Redox 1 bottle blood culture system, and PCR), either alone or in combination, in determining the causative agents in cases of endophthalmitis.

## Methods

### Patients and sample collection

Between February 2012 and February 2013, 41 specimens were collected from 29 cases of presumed acute endophthalmitis at King Chulalongkorn Memorial Hospital, Bangkok, Thailand. All patients who underwent a vitreous specimen collection were enrolled in this study. After information forms were given to patients and consent forms were signed, vitreous specimens were collected by three methods: pars plana vitrectomy, vitreous tapping, or evisceration. Methods were selected based on disease severity and the treatment plan for the patient.

Presumed acute endophthalmitis was defined as inflammation caused by a suspected bacterial infection. Symptoms included sudden loss of vision, ocular pain, photophobia, red eye, anterior chamber cells and flare, hypopyon, and vitreous cell clumping. The onset of symptoms was no longer than 6 weeks, and infection was associated with recent post-intraocular surgery, intraocular trauma, or endogenous infection. Patients with a history or final diagnosis of uveitis, those from whom the specimen obtained was inadequate for laboratory analysis, and those younger than 18 years were excluded from this study.

Demographic data and baseline characteristics were collected, including age, sex, underlying disease, history of eye disease, history of ocular surgery, history of ocular trauma, history of bacterial infection from other sources, onset of clinical symptoms, and history of previous treatment. An eye examination was performed that included best corrected visual acuity at presentation, intraocular pressure measurement, anterior chamber reaction and hypopyon, grading of vitreous cells and haze, B-scan ultrasound in case of poor vitreous visualization, and investigation of other, systemic sources of infection.

The vitreous specimens were collected in the operating theater at King Chulalongkorn Memorial Hospital. Each specimen was then divided into three parts, each comprising at least 0.1 ml. Each of these was then analyzed using one of three separate bacterial identification methods: conventional plate culture, a blood culture system, and PCR followed by gene sequencing. Multiple vitreous specimens from each patient were analyzed separately. Results from the three different identification methods were then compared.

Vitreous tapping was performed using 3-cc syringes with 23-G needles, with aspiration after insertion into the vitreous cavity to a depth of at least 1 cm. A 21-G needle was used if aspirate was not obtained after the first attempt. Pars plana vitrectomy was performed by retina fellows or retina staff. A sutureless 23-G and 25-G vitrectomy system was used. Following the placement of vitrectomy ports, vitreous fluid was aspirated using a vitrectomy cutter while the infusion system was turned off. For evisceration, specimens were directly aspirated into a 3-cc syringe after being exuded from the eyeball.

All participants were scheduled for follow-up visits at 1 week, 1 month, and 3 months post-operatively. Follow-up examination data were recorded at each visit.

### Techniques for bacterial identification

#### Conventional method

Conventional culture vitreous specimens were inoculated onto blood agar (tryptic soy agar (Oxoid, Basingstoke, UK) with 5% sheep blood); chocolate agar (Oxoid); *Brucella* agar (Becton Dickinson, San Jose, CA, USA) supplemented with 5% sheep blood, hemin, and vitamin K; and Sabouraud dextrose agar (Becton Dickinson); and into thioglycollate broth (Becton Dickinson). The blood agar and chocolate agar plates were incubated in 5% CO_2_ at 35 °C for 24–48 h. *Brucella* blood agar plates were incubated at 35 °C for 48–72 h, under anaerobic conditions achieved by placing each plate in an airtight bag or jar and using an anaerobic gas generator. Thioglycollate broth was incubated at 35 °C for 24–48 h. Sabouraud dextrose agar plates were incubated at 25 °C for a maximum of 30 days. The isolates were identified by conventional biochemical methods. Antimicrobial susceptibility testing was performed using the disk diffusion method and interpreted according to Clinical and Laboratory Standards Institute guidelines [20].

#### Blood culture system

VersaTREK Redox 1 bottles (TREK Diagnostic Systems, Cleveland, OH, USA) were used for aerobic blood culture in this study. The VersaTREK automated blood culture system uses aerobic bottles containing very small stir bars for continuous mixing of the sample in the broth. The bottles can be used for sample volumes of 0.1–1 ml. In this study, 0.1 ml of vitreous specimen was aspirated and injected directly into each VersaTREK Redox 1 bottle. Bottles were placed in an automated microbial detection drawer for a maximum of 5 days for detection of changes in gas production and consumption. This system can detect numerous aerobic microorganisms that produce or consume gas. After a positive signal was detected by the system, the positive broth sample was sub-cultured on blood agar, chocolate agar, and MacConkey agar (Oxoid) and incubated in 5% CO_2_ at 35 °C for 24–48 h. Resultant microorganisms were identified by conventional biochemical methods [20]. Antimicrobial susceptibility testing was carried out as described above. A final report for negative results was obtained within 5 days.

#### PCR analysis

Vitreous specimens obtained by vitreous tapping, vitrectomy and evisceration were aspirated and decanted into sterile microfuge tubes for PCR analysis of bacterial 16S rRNA genes [21]. Bacterial DNA was extracted using an ExiPrep Dx Bacteria Genomic DNA Kit (BIONEER, Seoul, Korea). Forward (5′-TGC CAG CAG CCG CGG TAA TAC-3′) and reverse (5′-CGC TCG TTG CGG GAC TTA ACC-3′) primers were used for 16S rRNA PCR amplification. The PCR mixture consisted of 1× PCR buffer, MgCl_2_ (2 mM), 200 μM each dNTP, 0.2 μM each primer, and 2 U of DNA polymerase (Faststart *Taq* DNA polymerase, Roche). The thermal cycling conditions used were as follows: 95 °C for 5 min; 35 cycles of 95 °C for 1 min, 55 °C for 1 min, and 72 °C for 1 min; then 72 °C for 10 min. The resultant amplicon was 593 bp. The PCR products were purified using a QIAquick PCR purification kit (Qiagen, Hilden, Germany). The purified PCR products were then sequenced using the chain termination method (Sanger sequencing) by 1^st^ Base (Kuala Lumpur, Malaysia). Nucleotide sequences were analyzed by BLAST analysis (http://blast.ncbi.nlm.nih.gov/) against the GenBank database and the Ribosomal Database Project.

### Sample size calculation and statistical analysis

The number of positive results obtained using each of the three techniques is reported as the percentage and mean. Cohen’s kappa coefficient test was used to identify agreement between sets of data (conventional plate culture, blood culture, and PCR). A *p-*value < 0.05 was considered statistically significant (R version 3.1.1, R Development Core Team, New Zealand).

## Results

Forty-one specimens were collected from 29 cases of presumed acute bacterial endophthalmitis. Demographic data, baseline characteristics, results of bacterial culture, and post-treatment visual recovery information are shown in Table 1. The patients included 16 females and 19 males, and the age range was 24–86 years (mean 57.03 years). Fifteen cases had a history of ocular surgery (37.5%), eight had a history of ocular trauma (20%), and six had evidence of other sources of infection (15%). In the post-operative group, a history of cataract surgery, pars plana vitrectomy, scleral buckle procedure, and trabeculectomy were present in 14 cases (93.3%), 3 cases (26.6%), 1 case (6.6%), and 1 case (6.6%), respectively. Twenty-four subjects presented with a best corrected visual acuity (BCVA) between counting fingers and light perception. Hypopyon was present in 14 of 29 cases (48.2%).

**Table 1:** Baseline characteristics, culture results, and follow-up data for individual subjects

Case	Endophthalmitis cause	Age	BCVA	Operation	Gram’s stain	C/S result	H/C result	PCR result	BCVA 1 week	BCVA 1 month	BCVA 3 months
1	trauma	44	HM with PJ	tap	*Neg rod*	N	*Streptococcus* Group D	N	S/P evisceration		
				evisceration	*Pos cocci*	N	*Streptococcus* Group D	*S. vestibularis*			
2	trauma	50	HM with PJ	tap	N	N	N	N	HM with PJ	20/40	20/20
				PPV	N	N	N	machine problem			
3	endogenous	46	20/70	PPV	N	N	N	machine problem	HM with PJ	NPL	NPL
4	post-operative	64	HM with PJ	tap	N	N	N	N	FC 1 ft	FC 4 ft	HM with PJ
5	post-operative	59	PL	PPV	N	N	N	*H. influenzae*	S/P evisceration		
				evisceration	N	N	N	*H. influenzae*			
6	endogenous	71	PJ	PPV	*N*	*Klebsiella pneumoniae*	*Klebsiella pneumoniae*	*Klebsiella pneumoniae*	S/P evisceration		
7	endogenous	72	HM with PJ	PPV	*Pos cocci*	*S. agalactiae*	*S. agalactiae*	*S. agalactiae*	S/P evisceration		
				tap	*N*	N	*S. agalactiae*	*S. agalactiae*			
				evisceration	Yeast	N	N	inconclusive			
8	trauma	71	HM with PJ	tap	*Neg rod*	*S. pneumonia*	N	N	S/P evisceration		
				evisceration	N	N	N	inconclusive			
9	post-operative	66	20/70	tap	N	N	Coag. neg. staphylococci	N	FC 1 ft	F/U other hospital	
10	post-operative	69	PJ	PPV	N	N	*E. faecalis*	*S. saccharolyticus E. faecalis*	PJ	HM with PJ	NPL
				tap	N	N	*E. faecalis*	*S. saccharolyticus E. faecalis*			
				PPV	N	N	*E. faecalis*	*S. saccharolyticus E. faecalis*			
11	post-operative	48	FC 1 ft	tap	N	N	N	N	20/30	20/20	loss F/U
				PPV	N	N	N	N			
12	post-operative	82	HM with PJ	PPV	N	N	N	N	FC 1/2 ft	FC 1/2 ft	PL
13	post-operative	74	5/200	PPV	N	N	N	*E. faecalis*	20/30	20/30	20/30
				tap	N	N	N	N			
14	post-operative	49	HM with PJ	PPV	Pos cocci	Coag. neg. staphylococci	Coag. neg. staphylococci	*S. haemolyticus* *S. simiae* *S. aureus*	FC 4 ft	20/200	20/200
				PPV	N	N	N	N			
15	trauma	47	HM with PJ	evisceration	N	N	N	N	Post-evisceration		
16	endogenous	34	HM with PJ	PPV	N	N	N	N	HM with PJ	HM with PJ	HM with PJ
17	post-operative	35	20/100	tap	N	N	N	N	20/50	F/U other hospital	
18	trauma	86	PL	tap	*Pos cocci*	*S. pneumonia*	*S. pneumonia*	N	Post-evisceration		
19	trauma	24	FC 1/2 ft	PPV	N	N	N	N	FC 1 ft	FC 1/2 ft	FC 1/2 ft
20	post-operative	49	HM with PJ	PPV	N	N	N	N	20/30	F/U other hospital	
21	endogenous	66	HM with PJ	tap	N	N	N	N	HM with PJ	HM with PJ	loss F/U
22	post-operative	50	FC 2 ft	tap	N	N	N	inconclusive	FC 4 ft	20/70	20/70
				PPV	N	N	N	N			
23	post-operative	72	FC 1 1/2 ft	tap	N	N	N	N	20/70	20/70	20/70
24	endogenous	46	HM with PJ	tap	N	N	N	N	Post-evisceration		
25	trauma	54	HM with PJ	PPV	N	N	N	N	Post-evisceration		
26	post-operative	64	FC 2 ft	PPV	Neg rod	N	N	N	FC 3 ft	FC 4 ft	FC 6 ft
27	post-operative	74	20/100	tap	N	N	N	N	FC 3 ft	20/200	20/100
28	post-operative	59	HM with PJ	tap	N	N	N	N	HM with PJ	FC ¼ ft	
29	trauma	29	PL	PPV	*Budding yeast*	*Curvularia*	N	N	HM with PJ		

*N*, no growth of bacteria; inconclusive, growth of multiple bacteria, unable to identified type of bacteria, or possible contamination; machine problem, no result due to machine error; *IOFB*, intraocular foreign body; phaco, phacoemulsification; *IOL*, intraocular lens implantation; *PPV*, pars plana vitrectomy; *ECCE*, extra-capsular cataract extraction; *SBP*, scleral buckling procedure; tap, vitreous tap; *C/S*, conventional bacterial culture method; *H/C*, blood culture bottle method; *PCR*, polymerase chain reaction method; *F/U*, follow up. *S.vestibularis, Streptococcus vestibularis; H. influenzae, Haemophilus influenzae; S. agalactiae, Streptococcus agalactiae; S. pneumoniae, Streptococcus pneumoniae,* Coag. neg. staphylococci*,* coagulase-negative staphylococci*; E. faecalis, Enterococcus faecalis; S. saccharolyticus, Staphylococcus saccharolyticus; S. haemolyticus, Staphylococcus haemolyticus; S. simiae, Staphylococcus simiae; S. aureus, Staphylococcus aureus;* HM, hand motion; PJ, light projection; NPL, no light perception; FC, counting finger; ft, feet; mo, month*; Pos cocci*, gram positive cocci*; Neg rod*, gram negative rod

The overall rate of positive bacterial identification in this study was 39%. In total, 12.2% of specimens were positively identified by conventional plate culture, 26.8% were identified by blood culture, and 26.8% were identified by PCR and sequencing. Conventional plate culture combined with blood culture had a positive bacterial identification rate of 29.3%, while conventional plate culture combined with PCR produced a positive identification rate of 31.7%. Blood culture combined with PCR had a positive identification rate of 34.1%. Of the 41 specimens, 19 were obtained by pars plana vitrectomy (47.5%), 17 by vitreous tapping (42.5%), and five by evisceration (12.5%). Rates of positive bacterial identification by technique are listed in Table 2.

**Table 2:** Bacterial identification based on specimen collection method

Type of specimen collection	CM	HC	PCR	Number of specimens negative by all methods (%)
Vitreous tap	n	*Strep gr. D*	n	4 (50)
	n	*S. agalactiae*	*S. agalactiae*	
	*S. pneumoniae*	n	n	
	n	*S. coagulase negative*	n	
	n	*E. faecalis*	*E. faecalis*	
	*S. pneumoniae*	*S. pneumoniae*	n	
	n	n	I	
PPV	n	n	*H. influenzae*	6 (31)
	*Klebsiella pneumoniae*	*Klebsiella pneumoniae*	*Klebsiella pneumoniae*	
	*S. agalactiae*	*S. agalactiae*	*S. agalactiae*	
	n	*E. faecalis*	*E. faecalis*	
	n	*E. faecalis*	*E. faecalis*	
	n	n	*E. faecalis*	
	*S. coagulase neg*	*S. coagulase neg*	*S. coagulase neg*	
Evisceration	n	*Strep gr. D*	*S. vestibularis*	3 (60)
	n	n	*H. influenzae*	
	n	n	I	
	n	n	I	

*CM*, conventional method; *HC*, hemoculture bottle method; *PCR*, polymerase chain reaction method; *n*, no growth; *I*, inconclusive; *Vitreous tap*, vitreous tapping; *PPV*, pars plana vitrectomy; *Strep gr. D*; *Streptococcus* Group D; *S. vestibularis, Streptococcus vestibularis; H. influenzae, Haemophilus influenzae; S. agalactiae, Streptococcus agalactiae; S. pneumoniae, Streptococcus pneumoniae; S* coagulase neg*,* Coagulase-negative staphylococci*; E. faecalis, Enterococcus faecalis*

BCVA at 1 week, 1 month, and 3 months post-operatively showed improvement of visual outcome (Table 1). Only one patient presenting with initial hand motion visual acuity achieved visual acuity of 20/20 at 3 months (case no. 2, Table 1).

Agreement analysis of the different bacterial identification methods determined by Cohen’s kappa test is shown in Table 3. There was a statistically significant agreement between the hemoculture bottle and PCR methods. However, there was no statistically significant agreement between the conventional plate culture method and either the hemoculture method or the PCR method.

**Table 3:** Analysis of agreement between bacterial culture methods

Methods	Kappa coefficient	95% CI	*P*-value
CM vs. HC	0.35	–0.03,0.72	0.057
CM vs. PCR	0.08	–0.27,0.42	0.33
HC vs. PCR	0.40	0.10,0.70	0.009

Estimate based on Cohen’s kappa coefficient and the test of the null hypothesis that the extent of agreement is the same as random (kappa = 0). *p*-value < 0.05 was considered statistically significant

*CM*, conventional method; *HC*, hemoculture bottle method; *PCR*, polymerase chain reaction method

## Discussion

In this study, positive bacterial identification was achieved for 12.2% of specimens using conventional culture methods, corresponding to a positive result for five of 29 cases (17.2%). This was slightly lower than previous reports (44.4–46%) [5, 6]. The present study included all cases of suspected acute bacterial endophthalmitis admitted to our hospital over the study period. This meant that some samples that would not usually be suitable for conventional culture analysis, including potentially contaminated samples and those from cases of severe uveitis. Another potential reason for the discrepancy is that we are a referral hospital and many cases had received treatment prior to admission to our hospital. Gram staining positively identified bacteria in approximately 17% of the cases examined; however, in 28% of cases the presence of Gram-positive bacteria was not associated with positive culture results. This might have been due to glass slide contamination. Visual acuity in all cases positive for Gram-positive cocci was below that required to distinguish hand motion, which indicates a high disease severity and number of bacteria. Previous antibiotic treatment can interfere with the bacterial identification rate. Our results show that blood culture and PCR analysis improved the rate of bacterial identification to 36.5% (15 of 41 specimens). Yospaiboon *et al.* [10] achieved a positive identification rate of 51.9% in vitreous fluid samples analyzed using blood culture bottles (BacT/ALERT), while Eser *et al.* [8] achieved a positive identification rate of 70.8%, again using blood culture bottles (Pediatric-Plus hemoculture bottle, Bactec), with *Staphylococcus epidermidis* being the most commonly identified microorganism (in 17.6% of samples). In the current study, VersaTrex Redox 1 bottles were used. This system is comparable with BacT/ALERT bottles, and exhibits significantly higher rates of detection in patients receiving antimicrobial therapy [22]. A study by Joseph *et al.* [23] had a positive detection rate of 66% using 16S rRNA PCR and sequencing. Real-time PCR can also improve rates of identification by 60–90% compared with conventional culture [13]. In addition, PCR detection of eubacterial genomes has been shown to have 100% correlation with positive specimens [17].

Melo *et al.* [5] studied the microbial profiles of patients diagnosed with suspected endophthalmitis and found that 91% of bacteria in such cases were Gram-positive. Among these cases, coagulase-negative staphylococci were the most common organisms (48%). Staphylococcal species are also the predominant cause of acute onset endophthalmitis [6]. In our study, vitreous specimens from only 10 of 29 cases were positive for bacterial microorganisms. Gram-positive microorganisms were mostly isolated in cases of acute presumed endophthalmitis (8 of 10 cases, 80%), which is comparable with the findings of previous studies [5, 6], and *Streptococcus* species were predominantly identified (4 of 10 cases, 40%). *Staphylococcus* and *Enterococcus species* were isolated in two cases, and *Klebsiella pneumoniae* and *Haemophilus* species were each found in only one case. The PCR approach identified additional microorganisms in eight specimens that were not identified by the conventional culture method. Four specimens were positive for *Enterococcus faecalis* by PCR; three of these cases were also positively identified by hemoculture. None of these specimens were identified by conventional culture methods. *E. faecalis* is a Gram-positive coccus that causes rapid progression of visual disturbance, but has a fair prognosis for positive outcome following treatment. It is a normal part of the flora of the gastrointestinal tract, but not of the ocular surface. Our results indicate that *E. faecalis* is a common pathogen in cases of endophthalmitis. The inability of conventional plate culture methods to identify *E. faecalis* may be explained by the ability of this pathogen to enter a viable but non-culturable (VBNC) state. The VBNC state is a survival strategy of bacteria when they are faced with hostile environmental conditions. *E. faecalis* in the VBNC state can still be detected by PCR [24].

PCR analysis identified *Haemophilus* species in two specimens, neither of which were detected by the conventional or blood culture methods. This might be explained by the fact that *Haemophilus* species are fastidious, and that the sample likely contained only small amounts of the bacteria, which could be missed by both culture techniques. A study has shown that even extensive subculturing and prolonged incubation of these bacteria to increase detection rates by standard automated blood culture does not significantly increase bacterial detection rates [25].

The PCR method used in the current study failed to detect *Streptococcus* species in two specimens and coagulase-negative staphylococci in one specimen (Table 2). This could be the result of the small volume of microorganisms collected, meaning that the number of cells was below the limit of detection. An unsuitable DNA extraction method for these organisms may also have inhibited their detection, or the presence of inhibitors could also have caused the amplification process to fail.

Blood culture medium helps to amplify the number of organisms in a hemoculture bottle prior to subculture on agar plates, meaning that theoretically it should improve rates of microbial detection in comparison with conventional culture techniques. In case no. 7 in our study, a positive identification was made by conventional plate culture but not by hemoculture or PCR. The identified organism was *Streptococcus pneumoniae*, which requires blood for growth. Blood agar is included in the conventional culture identification method, whereas neither the hemoculture nor the PCR technique used a blood-containing medium. Because there was little blood present in the vitreous specimen, only the conventional culture method identified *S. pneumoniae*. Interestingly, no positive identification was made of bacteria in evisceration specimens using culture methods, but PCR identified organisms in these samples. Evisceration is one of the final options for treatment of endophthalmitis. Most patients who underwent evisceration in the current study also underwent intravitreous antibiotic and/or systemic antibiotic treatment. Problems optimizing the PCR assay during the early stages of our study meant that results for two specimens are missing, and those for three specimens were inconclusive. This may be the result of a mixed culture or incorrect DNA extraction measures. To account for these issues, we analyzed the data by both substituting data for these samples with negative results, and by exclusion of the entire data sets for these five specimens. Neither of these analysis methods produced statistically significant results (*p* = 0.050).

The major limitations of this study are the small number of cases and the small volume of vitreous specimen collected, which was limited by the type of specimen. A greater number of specimens should be examined to better determine the differences between the methods, and other bacterial identification techniques, such as real-time PCR, may help to increase the rates of positive pathogen identification in endophthalmitis. Clinical correlation is important for diagnosis of infectious bacterial endophthalmitis. It is necessary to exercise caution when interpreting bacterial identification results, because positive identification of bacteria might relate to either environment contamination or a true bacterial pathogen.

## Conclusions

Based on our findings, blood culture bottles and PCR analysis can increase rates of bacterial identification in cases of presumed acute endophthalmitis. VersaTrex Redox 1 bottles were particularly useful for bacterial identification in vitreous specimens, while PCR increased the rates of detection for most bacteria, but particularly for *Haemophilus influenzae* and *E. faecalis*. Our findings suggest that blood culture bottles or PCR analysis should be added to standard diagnostic protocols in all cases of presumed bacterial endophthalmitis to increase the likelihood of a positive treatment outcome.

## Abbreviations

BCVA: Best corrected visual acuity
CM: Conventional method
HC: Hemoculture bottle
PCR: Polymerase chain reaction
VBNC: Viable but non-culturable
